# Screening and functional pathway analysis of genes associated with pediatric allergic asthma using a DNA microarray

**DOI:** 10.3892/mmr.2015.3277

**Published:** 2015-01-29

**Authors:** LI-QUN LU, WEI LIAO

**Affiliations:** 1 Department of Pediatrics, First Hospital Affiliated to Chengdu Medical College, Chengdu, Sichuan 610500, P.R. China; 2 Department of Pediatrics, Southwest Hospital, The Third Military Medical University, Chongqing 400038, P.R. China

**Keywords:** differentially expressed genes, functional enrichment, pathway analysis, pediatric allergic asthma

## Abstract

The present study aimed to identify differentially expressed genes (DEGs) associated with pediatric allergic asthma, and to analyze the functional pathways of the selected target genes, in order to explore the pathogenesis of the disease. The GSE18965 gene expression profile was downloaded from the Gene Expression Omnibus database and was preprocessed. This gene expression profile consisted of seven normal samples and nine samples from patients with pediatric allergic asthma. The DEGs between the normal and pediatric allergic asthma samples were screened using limma package in R, and the cut-off value was set at false discovery rate <0.05 and log fold change >1. Following hierarchical clustering of the DEGs based on the expression profiles, the up- and downregulated genes underwent a functional enrichment analysis by topological approach (P<0.05), using the Database for Annotation, Visualization and Integrated Discovery. A total of 127 DEGs were identified between the normal and pediatric allergic asthma samples. The up- and downregulated genes were significantly enriched in the actin filament-based process and the monosaccharide metabolic process, respectively. Seven downregulated DEGs (M6PR, TPP1, GLB1, NEU1, ACP2, LAMP1 and HGSNAT) were identified in the lysosomal pathway, with P=6.4×10^−9^. These results suggested that variation in lysosomal function, triggered by the seven downregulated genes, may lead to aberrant functioning of the T lymphocytes, resulting in asthma. Further research regarding the treatment of pediatric allergic asthma through targeting lysosomal function is required.

## Introduction

Allergic asthma is a complex respiratory disorder, which is characterized by airway inflammation, bronchial hyperresponsiveness and reversible airway obstruction (1). In recent decades, an increasing number of patients have been diagnosed with allergic asthma (2), and allergic diseases such as asthma have become a social problem that negatively affects the quality of life of sufferers. The incidence of allergic diseases, including asthma, has risen since the mid-20th century, with much of the increase associated with changes in the environment that affect the immune system (3). The exact mechanism for the progression of pediatric allergic diseases has yet to be elucidated; however, it appears to be a complex interaction between genetics, environmental exposure and sensitization (4). Previous studies have identified small molecular medicines, which may be used to treat allergic asthma in the future (5); however, adherence rates for asthmatic patients are problematic, ranging between 30 and 70% (6). Fewer than half of the patients treated with inhaled asthma medications adhere to their prescribed regimens (7), and the level of adherence is similar for children (8). Further molecular and genetic research is required to elucidate the underlying molecular mechanisms of allergic asthma.

Microarray DNA hybridization techniques are widely used in molecular biology research. In a DNA microarray, various DNA probes are immobilized onto a solid support in groups, forming an array of microspots. Hybridization to the microarray can then be performed by applying sample DNA solutions, either in bulk or in a microfluidic manner. Once the sample DNA has bound to the immobilized probe DNA through complementary sequence binding, detection is achieved through the read-out of the tagged markers attached to the sample target DNA (9). Genome-wide microarray studies of pooled DNA samples are a valuable tool, which may be used to identify candidate differentially expressed genes (DEGs) that are associated with a phenotype in a fast, scalable and economical manner (10). Previous studies has used microarray techniques and has reported changes in the expression of genes associated with viral transcription (RPL3, RPS10, RPL27, RPS11, RPL27A, RPL37A, EIF5A, EIF5B, and EEF1D) and lysosome function (ALAS1, ACO1, GPX3, PGD, VKORC1, and DCXR), which may be associated with the exacerbation of allergic asthma (5).

Investigating variations in gene expression, which can be quantitatively measured on a genome-wide scale, is essential for understanding and interpreting the pathogenic mechanism of pediatric allergic asthma. The present study used a DNA microarray method to identify the DEGs between normal and pediatric allergic asthma samples. The DEGs were then clustered. Functional and pathway analyses of the potential DEGs were then conducted, and the pathways were finally annotated based on the Kyoto Encyclopedia of Genomes and Genes (KEGG). The DEGs present in significant pathways associated with allergic asthma were further analyzed in order to explore the pathogenesis of the disease.

## Materials and methods

### Microarray data

The GSE18965 gene expression profile of pediatric allergic asthma (11) was downloaded from the public functional genomics data repository: The Gene Expression Omnibus (GEO; http://www.ncbi.nlm.nih.gov/geo/) (11). A total of 16 specimens, including seven normal samples and nine samples from patients with pediatric allergic asthma, were available. The gene expression profile was based on the platform of GPL96 (HG-U133A) Affymetrix Human Genome U133A Array (Affymetrix, Santa Clara, CA, USA).

### Data processing and identification of DEGs

Based on the annotation platform, 22,283 available probe IDs were mapped to gene names and 20,952 genes were selected and their expression profiles were processed (12). The limma package in R software was used to identify the DEGs between the normal and pediatric allergic asthma samples (13). The false discovery rate (FDR) was previously described by Benjamini and Hochberg (14), and is the expected proportion of false discoveries, out of the total number of identified DEGs. Applying a cut-off limit for FDR can help reduce error from multiplicity, whilst ensuring the identification of real DEGs. In the present study, cut-off values of log fold-change (|logFC|)>1.0 and an adjusted P<0.05 were used to identify DEGs.

### Hierarchical clustering of DEGs

Hierarchical clustering is a method used to build a hierarchy of clusters of DEGs. The process of clustering was based on the Euclidean distance (15) between the expression profiles of each of the DEGs filtered from the samples. The clustering was conducted using pheatmap (http://cran.r-project.org/web/packages/pheatmap/index.html) in R (16,17).

Strategies for hierarchical clustering generally fall into two categories. The ‘bottom up’ approach is where each DEG begins as a single cluster, and DEGs with similar expression profiles begin to successively merge as one cluster moves up the hierarchy. The ‘top down’ approach is where all DEGs begin as one cluster, and splits are performed recursively as a cluster moves down the hierarchy. Generally, the merges and splits are determined in a greedy manner. The results of hierarchical clustering are usually presented in a dendrogram.

### Functional enrichment analysis of DEGs

The Database for Annotation, Visualization and Integrated Discovery (DAVID; http://david.abcc.ncifcrf.gov/) (18,19) was then used to identify the enriched Gene Ontology (GO) biological processes that the up- and downregulated DEGs were associated with (P<0.05). The negative logarithmic P-values of each enrichment were displayed. The functional enrichments were presented in a bar chart, with the P-values displayed in a line chart, using Plotrix (http://cran.r-project.org/web/packages/plotrix/index.html) in R.

### Pathway analysis

Pathway enrichment analysis of all of the DEGs was performed using the KEGG database (http://www.kegg.jp/) (20). The KEGG maps of biological functions, and the corresponding DEGs were obtained.

## Results

### Data processing and identification of DEGs

Following normalization, a differential comparison between the expression profiles was performed, with the cut-off values set at FDR<0.05 and |logFC|>1. A total of 127 DEGs were identified, of which 58 were downregulated and 69 were upregulated (Table I).

### Hierarchical clustering of DEGs

**Figure 1:**
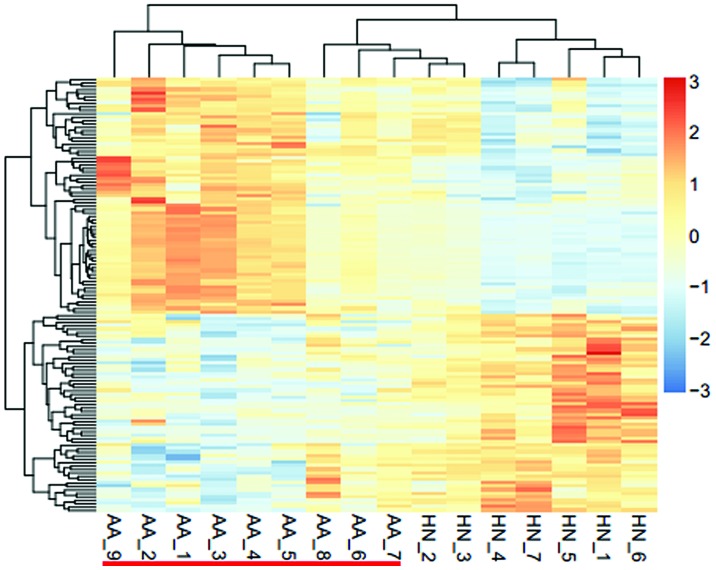
Clustering of differentially expressed genes using the hierarchical clustering method. Colors changing from blue to orange represent the differences in gene expression between the disease group and the healthy group, from downregulation to upregulation. Samples underlined red represent samples from the patients with pediatric allergic asthma. Samples displayed are all the ones in the dataset GSE18965. Each row represents a single gene; each column represents a tissue sample. AA, asthma atopic; HN, healthy non-atopic.

Hierarchical clustering of the identified genes is presented in Fig. 1. The logFC values of the DEGs ranged between three times upregulated and three times downregulated. The samples from the patients with pediatric allergic asthma could easily be distinguished from the samples of the healthy control group. These results suggested that the identified DEGs were significantly characteristic of allergic asthma and may be used to distinguish between normal samples and those from patients with asthma.

### Functional enrichment analysis of DEGs

**Figure 2:**
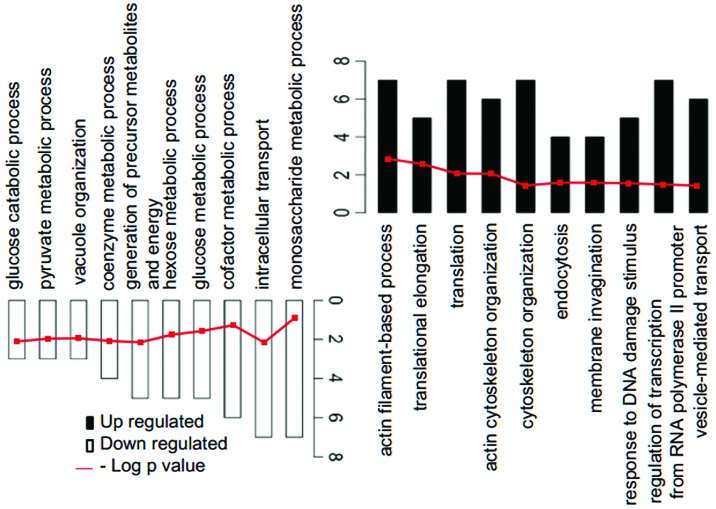
Functional enrichment of the up-and downregulated DEGs. Height of the bar represents the number of DEG enriched in the function nodes. The red line displays the negative logarithm of the P-values. White bars represent the functional enrichment of the downregulated DEGs, and black bars represent the upregulated DEGs. DEG, differentially expressed gene.

The identified DEGs were assembled into up- and downregulated genes and mapped into DAVID for functional enrichment analysis using the topological approach (Table I). The available functional enrichments are presented in a bar chart, with the P-values displayed in a line chart (Fig. 2). The up- and downregulated genes were significantly enriched in the actin filament-based process and the monosaccharide metabolic process, respectively.

### Pathway analysis

For further detail regarding the biological processes in which the identified DEGs participated in, part of these pathways were analyzed using KEGG. A pathway shown to be associated with the downregulated genes was the lysosomal pathway (P=6.4×10^−9^), of which seven downregulated DEGs were involved [M6PR, TPP1, GLB1, NEU1, ACP2, LAMP1 and HGSNAT].

## Discussion

Allergic asthma is characterized by airway hyperresponsiveness, inflammation and a cellular infiltration, which is dominated by eosinophils (21). Numerous epidemiological studies have linked the exacerbation of allergic asthma with an increase in ambient inhalable particulate matter from air pollutants (22,23). Furthermore, the majority of cases of allergic asthma have been attributed to infection with respiratory viruses, as well as other allergens (24). These infectious and allergic stimuli induce airway hyper-responsiveness by stimulating T lymphocytes and chemotaxis of acidophilic leukocytes (25), which results in the production of various pro-inflammatory cytokines and mediators to induce inflammation (26). The present study identified the up- and downregulated DEGs in allergic asthma, which were significantly enriched in the actin filament-based process and the monosaccharide metabolic process, respectively. Concordant with the findings of the present study, Wang *et al* (5) also demonstrated that the downregulated genes were associated with the monosaccharide metabolic process. Husain *et al* (27) previously reported that the actin filament-based process [GO:0030029] is enriched in food allergy, thus suggesting a possible involvement of certain DEGs with smooth muscle contraction, bronchoconstriction and vasodilation, which are common characteristics associated with type I allergic responses and anaphylaxis (27). One of the DEGs enriched in this process is scinderin (Scin), an actin-filament severing and capping protein that is activated by calcium (27). It has been suggested that Scin may be a potential biomarker of type I allergies, such as asthma (28). Furthermore, the upregulated genes (PDPK1, EZR, MYO6, CDC42BPA, OPHN1, ARF6 and WASL) identified in the present study that were enriched in the actin filament-based process require further study in order to determine whether they may be used as potential biomarkers of allergic asthma.

Viral transcription-associated proteins have previously been identified as DEGs in asthma (5). The present study identified seven downregulated DEGs (M6PR, TPP1, GLB1, NEU1, ACP2, LAMP1 and HGSNAT) that are associated with lysosomal function, which are associated with the autolysis of cells. A previous study demonstrated that the absence of MPRs and recycling cell surface receptors may lead to distinguishment of lysosomes, membrane-bound organelles that contain numerous hydrolytic enzymes from endosomes (29). TPP1 has previously been established as a shared or restricted regulatory dendritic cell (DC) marker (30), which has been suggested to have an important role in the development of atopic asthma (31). GLB1 gives rise to the GLB1 lysosomal enzyme and the elastin binding protein (EBP), which are involved in elastic fiber deposition (32). GLB1 forms a complex with protective protein cathepsin A (PPCA), NEU1 and galactosamine 6-sulphate sulfatase inside lysosomes, whereas EBP binds PPCA and NEU1 on the cell surface. ACP is present in the lysosomes inside DCs and was previously reported as a key enzyme that is able to digest antigens (33), thus indicating that it may have a similar role in allergic asthma. The specific functions of LAMP-1 and -2, which belong to the N-glycosylated proteins present in lysosomal membranes, have only recently begun to be recognized (34). The normal functions of LAMP-1 can be substituted by the structurally-associated LAMP-2; however, LAMP-2 has more specific tasks. Knockout of LAMP-2 expression in mice has revealed roles for LAMP-2 in lysosomal enzyme targeting, autophagy and lysosomal biogenesis (35). Furthermore, LAMP-2 deficiency in humans leads to Danon disease, which is associated with fatal cardiomyopathy and myopathy (36). A previous study demonstrated that loss of HGSNAT activity leads to mucopolysaccharidosis IIIC (MPSIIIC), a lysosomal disease (37). Subsequent ana lysis of this novel lysosomal protein revealed mutations in MPSIIIC and confirmed that it encoded HGSNAT (38). Among the genes identified in the present study (M6PR, TPP1, GLB1, NEU1, ACP2, LAMP1 and HGSNAT) that were significantly associated with lysosomal function, only TPP1 has been confirmed as being associated with atopic asthma (29). Further analyses of the specific functions of these identified DEGs in atopic asthma is required.

In conclusion, the results of the present study provide information on the underlying molecular mechanism of allergic asthma and provide a basis for future research. It was hypothesized that the identified downregulation of M6PR, TPP1, GLB1, NEU1, ACP2, LAMP1 and HGSNAT leads to disorders of lysosome function, which results in asthma by causing T-cell dysfunction. To date, the discovery of drugs for the treatment of pediatric asthma has been limited as its pathogenesis has yet to be fully elucidated. Therefore, the DEGs identified in the present study may provide a basis for the development of future medication used to treat this disease.

## Figures and Tables

**Figure 3:**
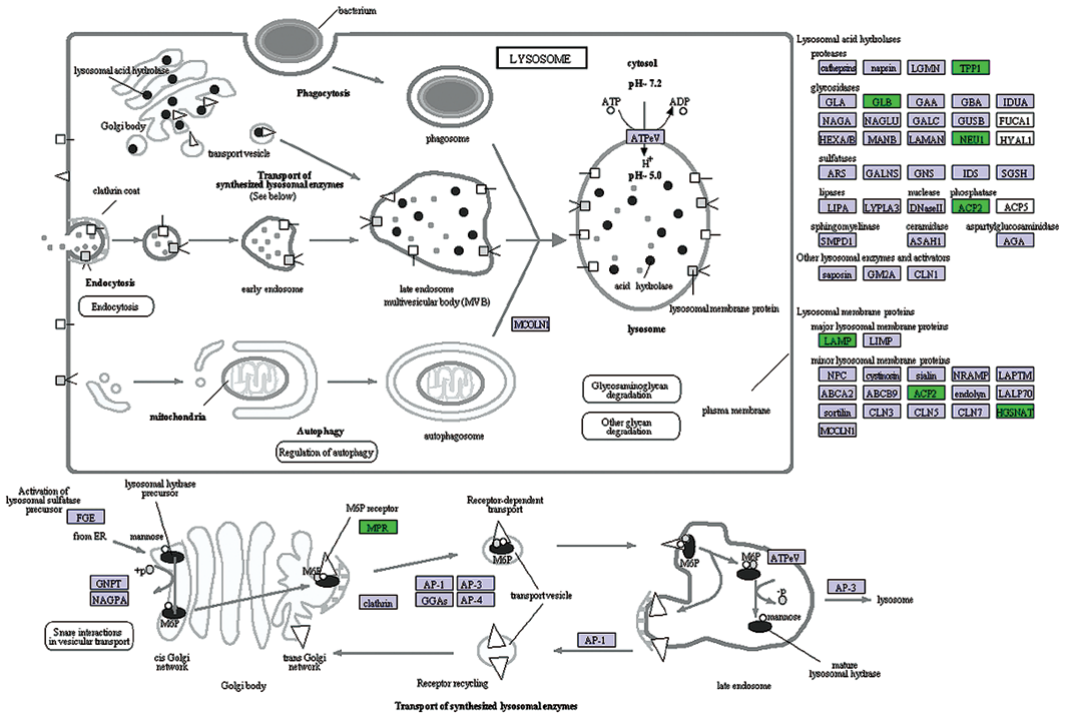
The lysosomal pathway is closely associated with the downregulated DEGs. Genes in green boxes represent the selected down-regulated DEGs. DEG, differentially expressed gene.

**Table I:** Differentially expressed genes in pediatric allergic asthma.

Genes	ID	adj.P-val	logFC
Downregulated genes			
GPI	208308_s_at	0.023	−1.44282
MLXIP	202519_at	0.023	−1.32908
TPP1	200742_s_at	0.023	−1.26717
NEU1	208926_at	0.023	−1.21158
ACTN1	208636_at	0.023	−1.20938
LAMP1	201551_s_at	0.040	−1.14585
MYOF	211864_s_at	0.027	−1.14455
GLB1	201576_s_at	0.037	−1.13821
NFKB1	209239_at	0.044	−1.13729
ACP2	202767_at	0.043	−1.08902
M6PR	200900_s_at	0.047	−1.06069
HGSNAT	218017_s_at	0.023	−1.05619
Upregulated genes			
UTP14A	221098_x_at	0.026	1.00982
KLHL23	213610_s_at	0.023	1.02244
EFCAB11	210525_x_at	0.023	1.02378
NUCKS1	217802_s_at	0.025	1.03147
FIP1L1	221007_s_at	0.023	1.03372
MBD4	214048_at	0.023	1.03480
PPFIBP1	203735_x_at	0.023	1.03706
INHBC	207688_s_at	0.026	1.04053
PPARA	210771_at	0.023	1.04131
EZR	217234_s_at	0.038	1.04663
CDC42BPA	214464_at	0.023	1.05413
INVS	211054_at	0.023	1.06234
BMP2K	37170_at	0.026	1.06702
RREB1	203704_s_at	0.045	1.07460
DMP1	217067_s_at	0.023	1.07594

logFC, log fold change; adj.P-val, adjusted P-value. |logFC|>1.0 and adj. P-val<0.05 were selected as the cut-off values for identification of differentially expressed genes.

**Table II:** Functional enrichment of the up- and downregulated differentially expressed genes in pediatric allergic asthma.

GO Term	Function	Count	P-value	Genes
Upregulated genes				
GO: 0030029	Actin filament-based process	7	4.20xl0^4^	PDPK1, EZR, MY06, CDC42BPA, OPHN1, ARF6, WASL
GO: 0006414	Translational elongation	5	7.45xl0^4^	RPL27A, RPL37A, RPS11, RPL38, EEF1D
GO: 0006412	Translation	7	0.002167	RPL27A, EIF5B, EIF5A, RPL37A, RPS11, RPL38, EEF1D
GO: 0030036	Actin cytoskeleton organization	6	0.002196	PDPK1, EZR, CDC42BPA, OPHN1, ARF6, WASL
GO: 0007010	Cytoskeleton organization	7	0.008267	PDPK1, EZR, CDC42BPA, OPHN1, ARF6, WASL, SMC3
GO: 0006897	Endocytosis	4	0.006008	MY06, FOLR1, OPHN1, EEA1
GO: 0010324	Membrane invagination	4	0.006008	MY06, FOLR1, OPHN1, EEA1
GO: 0006974	Response to DNA damage stimulus	5	0.006468	MY06, POLH, MBD4, FOX03, SMC3
GO: 0006357	Regulation of transcription from RNA polymerase II promoter	7	0.007357	IFNAR2, PPARA, ZBTB7A, CTBP1, MY06, FOX03, SMARCA2
GO: 0016192	Vesicle-mediated transport	6	0.008392	MY06, FOLR1, SEC22B, OPHN1, ARF6, EEA1
Downregulated genes				
GO: 0005996	Monosaccharide metabolic process	7	7.65xl0^5^	GPI, PGD, CHST4, GYG1, NAGK, DCXR, ENOl
GO: 0046907	Intracellular transport	7	0.018792	COPA, AP2B1, PSEN1, ATP6AP1, MLXIP, CTSA, M6PR
GO: 0051186	Cofactor metabolic process	6	4.07xl0^4^	ALAS 1, ACO1, GPX3, PGD, VKORC1, DCXR
GO: 0006006	Glucose metabolic process	5	0.001502	GPI, PGD, GYG1, DCXR, ENOl
GO: 0019318	Hexose metabolic process	5	0.003427	GPI, PGD, GYG1, DCXR, ENOl
GO: 0006091	Generation of precursor metabolites and energy	5	0.018456	GPI, ACO 1, ATP6AP1, GYG1, ENO1
GO: 0006732	Coenzyme metabolic process	4	0.013389	ACOl, GPX3, PGD, DCXR
GO: 0007033	Vacuole organization	3	0.007456	PSEN1,TPP1,ACP2
GO: 0006090	Pyruvate metabolic process	3	0.008197	SLC16A3,GPI,BSG
GO: 0006007	Glucose catabolic process	3	0.015228	GPI, PGD, ENOl

GO, gene ontology.

## References

[1] Tomita Y, Tomida S, Hasegawa Y (2004). Artificial neural network approach for selection of susceptible single nucleotide polymorphisms and construction of prediction model on childhood allergic asthma. BMC Bioinformatics.

[2] Mannino DM, Homa DM, Akinbami LJ, Moorman JE, Gwynn C, Redd SC (2002). Surveillance for asthma - United States, 1980–1999. MMWR Surveill Summ.

[3] Dietert RR, Zelikoff JT (2008). Early-life environment, developmental immunotoxicology, and the risk of pediatric allergic disease including asthma. Birth Defects Res B Dev Reprod Toxicol.

[4] Schmid-Ott G, Jaeger B, Adamek C (2001). Levels of circulating CD8(+) T lymphocytes, natural killer cells, and eosinophils increase upon acute psychosocial stress in patients with atopic dermatitis. J Allergy Clin Immunol.

[5] Wang XQ, Wang XM, Zhou TF, Dong LQ (2012). Screening of differentially expressed genes and small molecule drugs of pediatric allergic asthma with DNA microarray. Eur Rev Med Pharmacol Sci.

[6] Bender B, Milgrom H, Rand C (1997). Nonadherence in asthmatic patients: is there a solution to the problem?. Ann Allergy Asthma Immunol.

[7] Milgrom H, Bender B, Ackerson L, Bowry P, Smith B, Rand C (1996). Noncompliance and treatment failure in children with asthma. J Allergy Clin Immunol.

[8] Laird PW (2010). Principles and challenges of genomewide DNA methylation analysis. Nat Rev Genet.

[9] Creer TL, Wigal JK (1993). Self-efficacy. CHEST.

[10] Heller MJ (2002). DNA microarray technology: devices, systems, and applications. Annu Rev Biomed Eng.

[11] Kicic A, Hallstrand TS, Sutanto EN (2010). Decreased fibronectin production significantly contributes to dysregulated repair of asthmatic epithelium. Am J Respir Crit Care Med.

[12] Fujita A, Sato JR, Rodrigues LO, Ferreira CE, Sogayar MC (2006). Evaluating different methods of microarray data normalization. BMC Bioinformatics.

[13] Smyth GK, Gentleman R, Carey VJ, Huber W, Irizarry RA, Dudoit S (2005). limma: Linear models for microarray data. Bioinformatics and Computational Biology Solutions Using R and Bioconductor Statistics for Biology and Health.

[14] Benjamini Y, Hochberg Y (1995). Controlling the false discovery rate: a practical and powerful approach to multiple testing. J R Stat Soc Series B Stat Methodol.

[15] Deza MM, Deza E (2009). Encyclopedia of Distances.

[16] Szekely GJ, Rizzo ML (2005). Hierarchical clustering via joint between-within distances: Extending Ward’s minimum variance method. J Classif.

[17] Huson DH, Richter DC, Rausch C, Dezulian T, Franz M, Rupp R (2007). Dendroscope: An interactive viewer for large phylo-genetic trees. BMC Bioinformatics.

[18] Huang da W, Sherman BT, Lempicki RA (2009). Systematic and integrative analysis of large gene lists using DAVID bioinformatics resources. Nat Protoc.

[19] Huang da W, Sherman BT, Lempicki RA (2009). Bioinformatics enrichment tools: paths toward the comprehensive functional analysis of large gene lists. Nucleic Acids Res.

[20] Kanehisa M, Goto S, Hattori M (2006). From genomics to chemical genomics: new developments in KEGG. Nucleic Acids Res.

[21] Peden DB (2005). The epidemiology and genetics of asthma risk associated with air pollution. J Allergy Clin Immun.

[22] D’Amato G, Baena-Cagnani CE, Cecchi L (2013). Climate change, air pollution and extreme events leading to increasing prevalence of allergic respiratory diseases. Multidiscip Respir Med.

[23] Sugita M, Kuribayashi K, Nakagomi T, Miyata S, Matsuyama T, Kitada O (2003). Allergic bronchial asthma: airway inflammation and hyperresponsiveness. Intern Med.

[24] Yamaya M (2012). Virus infection-induced bronchial asthma exacerbation. Pulm Med.

[25] Docherty SJ, Butcher LM, Schalkwyk LC, Plomin R (2007). Applicability of DNA pools on 500 K SNP microarrays for cost-effective initial screens in genomewide association studies. BMC Genomics.

[26] Yasuda H, Suzuki T, Zayasu K (2005). Inflammatory and bronchospastic factors in asthma exacerbations caused by upper respiratory tract infections. Tohoku J Exp Med.

[27] Husain M, Boermans HJ, Karrow NA (2011). Mesenteric lymph node transcriptome profiles in BALB/c mice sensitized to three common food allergens. BMC Genomics.

[28] Di Valentin E, Crahay C, Garbacki N (2009). New asthma biomarkers: lessons from murine models of acute and chronic asthma. Am J Physiol Lung Cell Mol Physiol.

[29] Luzio JP, Rous BA, Bright NA, Pryor PR, Mullock BM, Piper RC (2000). Lysosome-endosome fusion and lysosome biogenesis. J Cell Sci.

[30] Zimmer A, Bouley J, Le Mignon M (2012). A regulatory dendritic cell signature correlates with the clinical efficacy of allergen-specific sublingual immunotherapy. J Allergy Clin Immunol.

[31] Liu Y, Liu S (2012). Protein-protein interaction network analysis of children atopic asthma. Eur Rev Med Pharmacol Sci.

[32] Caciotti A, Donati MA, Boneh A (2005). Role of beta-galactosidase and elastin binding protein in lysosomal and nonlysosomal complexes of patients with GM1-gangliosidosis. Hum Mutat.

[33] Hua H, Liang Z, Li W (2012). Phenotypic and functional maturation of murine dendritic cells (DCs) induced by purified Glycyrrhizin (GL). Int Immunopharmacol.

[34] Eskelinen EL (2006). Roles of LAMP-1 and LAMP-2 in lysosome biogenesis and autophagy. Mol Aspects Med.

[35] Tanaka Y, Guhde G, Suter A (2000). Accumulation of autophagic vacuoles and cardiomyopathy in LAMP-2-deficient mice. Nature.

[36] Eskelinen EL, Tanaka Y, Saftig P (2003). At the acidic edge: emerging functions for lysosomal membrane proteins. Trends Cell Biol.

[37] Ausseil J, Loredo-Osti JC, Verner A (2004). Localisation of a gene for mucopolysaccharidosis IIIC to the pericentromeric region of chromosome 8. J Med Genet.

[38] Fan X, Zhang H, Zhang S (2006). Identification of the gene encoding the enzyme deficient in mucopolysaccharidosis IIIC (Sanfilippo disease type C). Am J Hum Genet.

